# Application of the ICF based Norwegian function assessment scale to employees in Germany

**DOI:** 10.1186/s12995-017-0183-4

**Published:** 2018-01-11

**Authors:** Sylvia Jankowiak, Uwe Rose, Norbert Kersten

**Affiliations:** Division Work and Health, Federal Institute for Safety and Health (BAuA), Noeldnerstr. 40/42, D-10317 Berlin, Germany

**Keywords:** Occupational health, Employee health, International classification of functioning, disability and health, Surveys and questionnaires

## Abstract

**Background:**

At the interface of the occupational setting and rehabilitation, normative values for functional ability are desirable and worthwhile. The Norwegian Function Assessment Scale (NFAS) is a 39 item self-report instrument based on the International Classification of Functioning, Disability and Health (ICF). As the questionnaire was not used in a working population, we aimed to obtain functional levels of employees in Germany as measured through the NFAS.

**Methods:**

The NFAS was included in the Study on Mental Health at Work (S-MGA) 2011/12, a representative German survey of employees aged 31 to 60 years. For descriptive analyses, 95% confidence intervals were applied through bootstrap estimation to the skewed data of the NFAS (range from 1 = ‘no difficulty’ to 5 = ‘could not do it’). The data were analysed by age decades, professional qualification, and by disabilities, congenital diseases and accidents, stratified by sex. Linear regression analyses were conducted to estimate adjusted effects of age, professional qualification, and health limitations.

**Results:**

The NFAS total score was 1.17 (95% CI = 1.15–1.17). Thirty-five percent of the employees’ (1378 out of 3937 participants) reported the best possible functional ability (NFAS total score of 1.00). Managing and walking/standing were the NFAS’ most affected domains with a score of 1.26 (95% CI = 1.23–1.27), respectively. The regression analysis confirmed more functional difficulties for elder employees, females, employees with low professional qualification, and for employees suffering from disability and accidents.

**Conclusions:**

The study presents normative values of functional ability for the workforce. The results are useful for score interpretation in rehabilitation and return-to-work processes.

**Electronic supplementary material:**

The online version of this article (10.1186/s12995-017-0183-4) contains supplementary material, which is available to authorized users.

## Background

Functional ability is the actual or potential capacity of an individual to perform the activities and tasks that are common in daily life [1]. Functional ability is a key factor in determining an individual’s quality of life, and an important asset for both employees and the society [2]. National and international reports based on assessments by the respective social insurance systems revealed that a considerable proportion of the population suffers from reduced capacities for workforce participation [3, 4]. The social security systems are ultimately required to make the decision on disability pension applications. In Norway, in 2012, the percentage of disability pensioners in the population age group of 18–66 years was 9.5% [5]. In Germany, at the same time, the percentage of disability pensioners in the same age group was only 5.1% [6], and the Norwegian share also exceeds that of most other European countries [5]. In recent years, European social security schemes began to target individual capabilities rather than health deficits, first and foremost in the Nordic countries [7, 8]. In this context, new questionnaires have been developed, such as the Norwegian Function Assessment Scale, NFAS.

To date, the standard framework for describing and organising information on functioning and disability is the International Classification of Functioning, Disability and Health (ICF). The NFAS is a 39-item self-report questionnaire whose underpinning goes back to the ICF [9]. It aims to assess the functioning based on the individual’s self-perception, and was developed in 2000 by an expert group in social insurance [10]. Therefore, all categories from the activities/participation component of the ICF were considered. The expert group removed all categories that were not trusted to be relevant for the assessment of work-related functional abilities, resulting in a final list of 39 categories, which were then reformulated into questions [10].

To our knowledge, the NFAS has so far been applied in five studies, all of them conducted in Norway. Attention was drawn to sick-listed participants (*n* = 386) who showed a considerably reduced functioning [10] and to a sample of the general population aged 24 to 86 years (*n* = 1620) for obtaining normative data [11]. Furthermore, an association of NFAS values with a positive expectancy for eventually returning to work was demonstrated for disability pensioners with back pain (*n* = 89) [12]. The NFAS was also found to be a strong predictor of receiving allowances 3 years after an occupational rehabilitation due to long-term sick leave (*n* = 338) [13].

In all of these studies, a four-point scale was used for coding the responses. A trial study with a sample of the general population aged 24 to 86 years (*n* = 1705) revealed a clear superiority of a five- over a four-point scale, followed by the recommendation to use the more differentiated one in future studies [14].

The working population – a group the NFAS was also designed for – has as yet not been examined. However, an application to the working population would provide important normative data necessary for score interpretation and for defining objectives in rehabilitation. Therefore, in the Study on Mental Health at Work, S-MGA (Studie zur mentalen Gesundheit bei der Arbeit) [15] we set out to obtain normative data on the employed population in Germany as measured through the NFAS.

Germany’s workforce is composed of three groups: employees, civil servants, and persons engaged in freelancing or self-employment [16]. The term ‘employee’ includes the entire spectrum of sectors and the full range of requirements (working tasks from very simple to highly complex). In 2011, employees accounted for 81.4% of the workforce in Germany [16]. According to German law, employees have a liability for social security contributions (such as health, nursing, pension and unemployment insurance). The German Federal Employment Agency runs the register ‘Integrated Employment Biographies’, which solely focuses on the employees in Germany. Civil-servants and freelancers/self-employed persons (4.9% and 13.6% in 2011, respectively) are not registered, and they are obliged to pay for their health insurance only.

The purpose of this study was to obtain normative values on the NFAS as part of the Study on Mental Health at Work. In order to examine the variability of the functional ability depending on sex, age, professional qualification, disabilities, congenital diseases, and accidents we describe and estimate the influence of these factors on the NFAS scores.

## Methods

The Study on Mental Health at Work (S-MGA) is a nationwide panel study of employees in Germany with a five-year retake period [17]. The first assessment was conducted from November 2011 to June 2012 (S-MGA I) on a sample of 4511 employees. For S-MGA I employees aged 31 to 60 years were considered, because most Germans have completed their professional training/studies at this stage of their life and are not yet superannuated. The S-MGA serves two purposes: to examine the impact of employment on mental health and to analyse the association between functioning and work ability. The latter purpose constitutes the line of the present study.

### S-MGA I recruitment and study procedure

The population considered for the S-MGA I comprised the birth cohorts 1951 to 1980 who were subject to social insurance contributions in Germany on December 31st 2010. At this time, Germany had 31.9 million employees [18], and 21.5 million of them were aged 31 to 60 years [16, 17]. For creating a representative sample, a two-stage cluster sampling method was used [15]. Initially, all of the 12,227 German municipalities were stratified proportionally, by region and population size. Out of these, 206 municipalities throughout Germany were randomly selected. Next, the random gross sample was built. Therefore, 13,590 individuals were drawn within the randomly selected municipalities. One week prior to the first contact attempt, a letter including information on the study was sent to the registered addresses of the selected individuals. The survey was carried out by 243 trained interviewers on the condition that the respondent was fluent in German. The employees were interviewed face-to-face at their homes via computer-assisted personal interview (CAPI). The interviews took around 60 to 70 min to complete and covered socio-demographic information, employment and working conditions, work ability and functioning, motivational, volitional and personal co-factors, and mental health [17]. The obtained data was linked to the register ‘Integrated Employment Biographies’ if the participants gave their written permission. The full protocol of the study has been published elsewhere [17]. The four thousand, five hundred and eleven participants of the S-MGA I constitute a representative sample as it properly displays a variety of characteristics of employees in Germany, such as sex, education, employment, occupational status, working hours, nationality, and wages. For the present study, we included employees with a current weekly working time of at least 14 h per week, resulting in a final number of 3937 employees.

### The Norwegian function assessment scale (NFAS)

The NFAS consists of 39 items relevant for assessing the physical and mental functioning in the working life and in daily living. Based on a principal component analysis, the items are arranged across seven domains [10]. The physical domains are Walking/standing, Holding/picking up things, Lifting/carrying and Sitting; the mental domains cover Managing, Cooperation/communication and Senses. Each item is preceded by the following question: ‘Have you had difficulty doing the following activities during the last week ...?’ The NFAS was developed with a four-point scale [10] and is now recommended to be used with a five-point scale. Additionally, the original NFAS contains a ‘missing data’ category.

The German version of the NFAS was included in the S-MGA I [17], covering the same 39 items and its possible difficulty within the last week. The German NFAS version was generated in a five-step process. Firstly, the NFAS was translated by a bilingual Norwegian-German. Secondly, this translation was compared to the English NFAS version (as rated by four persons). Thirdly, a preliminary German NFAS was chosen. Next, it was back-translated by another bilingual person. This Norwegian back-translation was sent to the NFAS authors and users S. Brage and N. Østerås who found it remarkably similar (e-mail communication). As a last step, the very small changes suggested by the authors were adapted, and the final German NFAS was formed. As recommended by Østerås et al. [14] the German NFAS was applied by using a five-point scale: 1 = no difficulty; 2 = little difficulty; 3 = moderate difficulty; 4 = much difficulty; and 5 = could not do it. The German version aimed at differentiating the reasons for having missing items, and therefore three ‘missing data’ categories were applied: ‘inapplicable to me’, ‘refused’ and ‘don’t know’. The German version of the NFAS is provided as supplementary material [see Additional file 1].

### Variable selection

The basic confounder sex was dealt with stratification. Of the factors that potentially affect a person’s working life we chose the following variables as described in [3]: age, professional qualification, and the three health limitations disabilities, congenital diseases, and accidents.

Age was taken in decades, i.e. 31 to 40, 41 to 50, and 51–60 years. Professional qualification was assessed by using the three groups ‘university’, ‘occupational training’, and ‘unskilled/semi-skilled’. The group ‘university’ includes qualification from a university or a university of applied sciences; the group ‘occupational training’ considered having completed i) a training; ii) a vocational or commercial school; and iii) a master or technical college.

To form the binary variable ‘disabilities’, the existence of an official confirmation of the individuals ‘severely handicapped’ status was verified. Additionally, the degree of disability was recorded (range 20% to 100%). The binary variables ‘congenital diseases’ and ‘accidents’ had to be determined by a physician. The kind of congenital disease was not enquired. Accidental injuries (e.g. to the back, limbs, or burns) refer to the life time. For reasons of readability these three conditions were labeled ‘health limitations’.

### Data analysis

The seven domain scores were calculated by adding up the item scores and dividing by the number of items completed. At least 50 % of the items had to be answered to calculate any particular NFAS domain. The NFAS total scores were calculated by adding all 39 item scores and dividing by the number of items completed given that none of the domain scores was missing. Missing data were calculated as percentages and displayed for the category ‘inapplicable to me’. The selection of the scale’s bottom value (i.e. value ‘1’ no difficulty) was calculated as percentage per item as well.

For descriptive statistics we included the means of the item values, of the domain scores and of the total score. A low score indicates good functional ability. As we hypothesised differences in the NFAS scores for women and men, we ran stratified analyses. All tables are split by sex. Additionally, total values are given in the descriptive part.

The Norwegian studies [10, 11, 14] provided evidence that the distribution of item and domain scores is skewed to the right. Instead of providing standard deviations (SDs) for skewed data we computed 95% confidence intervals (CIs) through bootstrap estimation [19]. For this purpose a bias-corrected and accelerated method was used [19].

It was hypothesised that both the NFAS domain scores and the NFAS total score are influenced by age, professional qualification as well as disabilities, congenital disorders and accidents. Descriptive analyses show NFAS domain values with 95% CIs for each of the five factors.

For the comparison of means we used the fact that there is a very close relationship between confidence intervals and significance tests [20]. This approach uses the two separate CIs of both means to compare (as opposed to the CI of the difference of means). A conservative variant of a usual significant difference is indicated lest the CIs do not overlap. Here, the real α in probability is smaller than the nominal α of 0.05, even though the exact α is not provided.

To simultaneously estimate the partial effect of these five factors on the NFAS total score, generalised linear regression analyses were run (score value minus 1). The Tweedie function [21] served as distribution function because it covers the data’s specific distribution in an appropriate manner (link function identity, initial value *p* = 1.5). Tweedie’s compound Poisson distribution is a mixture of a degenerate distribution at zero and a continuous distribution on the positive real line [21]. All analyses were performed using the SPSS Statistics 23 software except for the bootstrap estimations which were calculated with SYSTAT 13.

## Results

The results were based on *n* = 3937 employees in Germany with a working time of at least 14 h per week. The employees’ mean (SD) age was 45.1 (4.5) years. The group age distribution was as follows: 31–40 years 21.3%, 41–50 years 39.0%, and 51–60 years 39.7%. The sample consisted of 47.5% women. Table 1 summarises the characteristics of the population.

**Table 1:** Characteristics of the study population

	Number	Percent
Full Sample	3937	100
Sex		
Women	1872	47.5
Men	2065	52.5
Age (years)		
51–60	1530	38.9
41–50	1589	40.4
31–40	818	20.8
Professional Qualification		
University	864	21.9
Occupational training	2870	72.9
Unskilled/semi-skilled	202	5.1
Health Limitations		
Disabilities	336	8.5
Congenital diseases	73	1.9
Accidents	1186	30.1

### Distribution and data quality

Table 2 depicts the percentage of inapplicability per NFAS item (column 2), the bottom value ‘1’ per item (column 3), and the mean NFAS domain and item scores (columns 4 to 9).

**Table 2:** Missing data, percentage of employees without difficulty and mean NFAS domain and item scores with 95% CI for the full sample and stratified by sex (*n* = 3937). A value of 1 characterises the best possible functional ability

	Full Sample (n = 3937)				Women (*n* = 1872)		Men (*n* = 2065)	
Domains/items^a^	ia^b^ (%)	nd^c^ (%)	${\overline{\mathrm{x}}}_T$	95% CI	${\overline{\mathrm{x}}}_W$	95% CI	${\overline{\mathrm{x}}}_M$	95% CI
**Walking/Standing**	**0.7**	**65.7**	**1.26**	**1.23–1.27**	**1.28**	**1.24–1.30**	**1.24**	**1.20–1.25**
01 Standing	0.1	82.2	1.31	1.27–1.33	1.35	1.29–1.37	1.28	1.23–1.31
02 Walking less than a kilometre on flat ground	0.3	89.6	1.19	1.16–1.20	1.21	1.17–1.23	1.17	1.13–1.19
03 Walking more than a kilometre on flat ground	1.5	84.7	1.27	1.23–1.29	1.32	1.26–1.35	1.23	1.19–1.26
04 Walking on different surfaces	0.8	85.3	1.26	1.23–1.28	1.30	1.25–1.33	1.23	1.19–1.25
05 Going up and down stairs	0.2	75.2	1.41	1.37–1.43	1.44	1.39–1.47	1.39	1.34–1.41
06 Going shopping for your groceries	2.2	87.4	1.18	1.15–1.19	1.22	1.17–1.24	1.14	1.11–1.16
07 Putting on your shoes and socks	0.0	89.4	1.18	1.15–1.19	1.16	1.12–1.18	1.19	1.15–1.21
**Holding/Picking up things**	**1.7**	**80.8**	**1.09**	**1.08–1.10**	**1.11**	**1.09–1.12**	**1.08**	**1.06–1.09**
08 Picking up a coin from a table with your fingers	0.6	95.7	1.06	1.04–1.07	1.08	1.05–1.09	1.04	1.03–1.05
09 Holding and turning a steering wheel	3.0	94.9	1.04	1.03–1.05	1.05	1.03–1.07	1.03	1.02–1.04
10 Driving a car	4.9	92.1	1.06	1.04–1.07	1.07	1.05–1.09	1.05	1.03–1.06
11 Preparing food	3.0	94.2	1.05	1.04–1.05	1.06	1.04–1.08	1.03	1.02–1.04
12 Writing	0.2	96.2	1.05	1.04–1.06	1.07	1.05–1.08	1.04	1.02–1.05
13 Performing everyday tasks on your own	0.1	92.8	1.10	1.08–1.11	1.13	1.11–1.15	1.08	1.05–1.09
14 Engaging in your leisure activities	1.4	84.6	1.26	1.22–1.28	1.28	1.23–1.31	1.24	1.20–1.27
15 Putting on and taking off your clothes	0.0	93.6	1.10	1.08–1.11	1.10	1.07–1.11	1.10	1.07–1.11
**Lifting/Carrying**	**4.0**	**75.6**	**1.16**	**1.14–1.17**	**1.20**	**1.17–1.21**	**1.12**	**1.09–1.13**
16 Lifting an empty soda bottle crate from the floor	0.8	87.9	1.20	1.17–1.22	1.28	1.23–1.30	1.14	1.10–1.16
17 Carrying shopping bags in your hands	1.4	86.8	1.21	1.18–1.22	1.29	1.25–1.32	1.13	1.10–1.15
18 Carrying a little sack/backpack on your shoulders or back	2.8	87.3	1.17	1.14–1.18	1.23	1.17–1.25	1.11	1.08–1.13
19 Pushing and pulling with your arms	0.5	88.7	1.19	1.16–1.20	1.22	1.18–1.25	1.15	1.11–1.17
20 Cleaning your house	7.1	83.0	1.18	1.15–1.19	1.24	1.20–1.27	1.11	1.08–1.12
21 Washing your clothes	11.2	83.6	1.09	1.07–1.10	1.11	1.08–1.12	1.06	1.04–1.08
**Sitting**	**6.5**	**88.6**	**1.11**	**1.09–1.12**	**1.13**	**1.10–1.14**	**1.10**	**1.08–1.11**
22 Sitting on a kitchen chair	0.3	93.3	1.10	1.08–1.11	1.12	1.11–1.14	1.08	1.06–1.09
23 Riding as a passenger in a car	2.8	89.9	1.13	1.10–1.14	1.14	1.11–1.16	1.11	1.08–1.13
24 Riding as a passenger on public transport	16.5	79.5	1.09	1.06–1.10	1.10	1.11–1.12	1.07	1.05–1.09
**Managing**	**2.2**	**51.4**	**1.26**	**1.23–1.27**	**1.29**	**1.26–1.30**	**1.23**	**1.21–1.24**
25 Staying alert and being able to concentrate	0.0	79.9	1.27	1.24–1.28	1.30	1.26–1.32	1.24	1.21–1.25
26 Working in groups	4.5	87.9	1.11	1.09–1.12	1.13	1.10–1.14	1.10	1.08–1.12
27 Guiding others in their activities	7.3	84.1	1.12	1.10–1.14	1.14	1.11–1.16	1.11	1.09–1.13
28 Managing everyday responsibility	0.1	87.7	1.17	1.15–1.18	1.20	1.17–1.22	1.14	1.11–1.16
29 Managing everyday stress and strains	0.1	79.1	1.28	1.25–1.30	1.32	1.27–1.34	1.24	1.21–1.26
30 Managing to take criticism	1.1	68.7	1.42	1.38–1.44	1.47	1.43–1.50	1.37	1.33–1.40
31 Managing to control your anger and aggression	2.1	69.5	1.38	1.35–1.40	1.39	1.35–1.42	1.37	1.32–1.39
**Cooperation/Communication**	**0.1**	**69.2**	**1.13**	**1.11–1.13**	**1.12**	**1.11–1.13**	**1.13**	**1.11–1.14**
32 Remembering things	0.0	74.6	1.33	1.30–1.34	1.34	1.29–1.36	1.32	1.28–1.34
33 Understanding spoken messages	0.0	89.2	1.13	1.11–1.14	1.12	1.10–1.14	1.13	1.11–1.14
34 Understanding written messages	0.1	90.9	1.11	1.09–1.12	1.11	1.09–1.12	1.11	1.09–1.12
35 Speaking	0.0	95.8	1.05	1.04–1.05	1.05	1.03–1.05	1.05	1.04–1.06
36 Participating in a conversation with many people	0.3	91.1	1.12	1.10–1.12	1.11	1.09–1.13	1.12	1.09–1.13
37 Using the telephone	0.0	97.3	1.04	1.02–1.04	1.03	1.02–1.04	1.04	1.03–1.05
**Senses**	**0.8**	**97.2**	**1.03**	**1.02–1.04**	**1.03**	**1.02–1.04**	**1.02**	**1.01–1.03**
38 Watching television	0.9	96.8	1.03	1.02–1.03	1.04	1.02–1.05	1.03	1.02–1.03
39 Listening to the radio	0.7	97.7	1.02	1.01–1.03	1.03	1.01–1.03	1.02	1.01–1.03
**Total**	**2.0**	**35.1**	**1.17**	**1.15–1.17**	**1.19**	**1.17–1.20**	**1.15**	**1.13–1.16**

^a^Domains are depicted in bold. Items are based on a five-point scale with 1 = ‘no difficulty’, 2 = ‘little difficulty’, 3 = ‘moderate difficulty’, 4 = ‘much difficulty’ and 5 = ‘could not do it’

^b^ia = inapplicable to me, ^c^nd = no difficulty

For each of the 39 items, the most frequent response was ‘no difficulty’ (69.5% to 98.4% of the answers per item). This resulted in an overall right-skewed distribution. Thirty-five per cent of the employees (33.0% of the women and 37.0% of the men) reported not a single difficulty on the whole questionnaire (only ‘1’s on a five-point scale). In contrast, six abilities were affected for at least every fifth employee, i.e. ‘going up and down stairs’ (item no. 05; domain Walking/standing), ‘remembering things’ (item no. 32; domain Cooperation/communication), and four items of the domain Managing: ‘staying alert and being able to concentrate’, ‘managing everyday stress and strains’, ‘managing to take criticism’, and ‘managing to control your anger and aggression’ (items no. 25, 29, 30, 31; Table 2).

Among employees in Germany the NFAS total score was ${\overline{\mathrm{x}}}_T$=1.17, CI = 1.15–1.17. Most severely affected was the ability to take criticism ${\overline{\mathrm{x}}}_T$=1.42, CI = 1.38–1.44, least affected the ability to listen to the radio ${\overline{\mathrm{x}}}_T$=1.02, CI = 1.01–1.02; Table 2. On the domains Managing and Walking/standing employees scored equally problematic with ${\overline{\mathrm{x}}}_T$=1.26, CI = 1.23–1.27. Least difficulty was reported for the NFAS domain Senses with ${\overline{\mathrm{x}}}_T$=1.03, CI = 1.02–1.04.

In general, men reported a significantly better functional ability than women (total score men ${\overline{\mathrm{x}}}_M$ =1.15, CI = 1.13–1.16 vs. women ${\overline{\mathrm{x}}}_W$ =1.19, CI = 1.17–1.20, Table 2). The in-depth results presented in Table 2 revealed 31 items where men scored better than women, apart from the eight items of the domains Cooperation/communication and Senses.

Almost all missing data (99.9%) were missing due to the fact that the activities in question were not applicable to the employees. On average, 2% of the NFAS values were not applicable. Predominant sectors included using public transport (item no. 24) and doing the laundry by oneself (item no. 21) with 16.5% and 11.2% of unconcerned employees, respectively (Table 2).

### Separate effects of age, professional education and health limitations on the NFAS

Table 3 shows the mean values of the domain scores and the total score in association to age, professional education and health limitations, stratified by sex.

**Table 3:** Mean NFAS domain and total scores with 95% CI (in parenthesis) by age decades, professional qualification, and for employees with disabilities, congenital diseases and accidents; stratified by sex (n = 1872 women and n = 2065 men). A value of 1 characterises the best possible functional ability

	Walking/standing	Holding/picking up things	Lifting/carrying	Sitting	Managing	Cooperation/communication	Senses	Total scores
Women								
Age (years)								
51–60 (*n* = 778)	1.39 (1.32–1.42)	1.13 (1.10–1.15)	1.26 (1.20–1.29)	1.17 (1.12–1.20)	1.30 (1.23–1.32)	1.15 (1.12–1.17)	1.03 (1.02–1.04)	1.23 (1.19–1.25)
41–50 (*n* = 744)	1.21 (1.16–1.25)	1.09 (1.06–1.11)	1.16 (1.12–1.18)	1.10 (1.06–1.12)	1.28 (1.24–1.31)	1.11 (1.09–1.13)	1.04 (1.02–1.05)	1.16 (1.13–1.18)
31–40 (*n* = 350)	1.21 (1.13–1.25)	1.09 (1.05–1.10)	1.14 (1.09–1.16)	1.11 (1.06–1.13)	1.27 (1.19–1.30)	1.10 (1.06–1.11)	1.02 (1.00–1.03)	1.15 (1.11–1.17)
Professional qualification								
Unskilled/semi-skilled (*n* = 97)	1.53 (1.32–1.66)	1.18 (1.07–1.25)	1.32 (1.17–1.42)	1.15 (1.04–1.24)	1.33 (1.19–1.41)	1.15 (1.08–1.20)	1.03 (1.01–1.05)	1.29 (1.19–1.36)
Occupational training (*n* = 1378)	1.30 (1.25–1.32)	1.11 (1.08–1.12)	1.21 (1.16–1.22)	1.14 (1.11–1.16)	1.29 (1.25–1.30)	1.13 (1.11–1.14)	1.04 (1.02–1.04)	1.20 (1.17–1.20)
University (*n* = 396)	1.18 (1.12–1.22)	1.09 (1.05–1.11)	1.14 (1.09–1.18)	1.07 (1.04–1.10)	1.27 (1.22–1.31)	1.12 (1.09–1.14)	1.01 (1.00–1.03)	1.15 (1.11–1.17)
Disabilities								
Cases (*n* = 148)	1.80 (1.62–1.91)	1.29 (1.19–1.34)	1.58 (1.41–1.68)	1.39 (1.24–1.48)	1.55 (1.41–1.63)	1.22 (1.14–1.26)	1.08 (1.02–1.12)	1.47 (1.37–1.53)
Non-cases (*n* = 1708)	1.23 (1.19–1.25)	1.08 (1.07–1.10)	1.16 (1.13–1.17)	1.10 (1.08–1.11)	1.26 (1.23–1.27)	1.12 (1.10–1.13)	1.03 (1.02–1.03)	1.16 (1.14–1.17)
Congenital Diseases								
Cases (*n* = 79)	1.52 (1.32–1.63)	1.20 (1.10–1.26)	1.39 (1.18–1.51)	1.24 (1.08–1.36)	1.44 (1.27–1.55)	1.13 (1.06–1.18)	1.07 (1.00–1.15)	1.33 (1.20–1.39)
Non-cases (*n* = 1791)	1.27 (1.22–1.29)	1.10 (1.08–1.11)	1.19 (1.16–1.20)	1.12 (1.10–1.14)	1.28 (1.25–1.29)	1.12 (1.11–1.13)	1.03 (1.02–1.04)	1.18 (1.16–1.19)
Accidents								
Cases (*n* = 414)	1.38 (1.30–1.44)	1.17 (1.12–1.20)	1.27 (1.20–1.31)	1.17 (1.10–1.20)	1.34 (1.29–1.38)	1.16 (1.12–1.18)	1.06 (1.02–1.08)	1.25 (1.21–1.28)
Non-cases (*n* = 1457)	1.26 (1.21–1.28)	1.09 (1.07–1.10)	1.18 (1.14–1.20)	1.12 (1.09–1.13)	1.27 (1.24–1.29)	1.12 (1.10–1.13)	1.02 (1.01–1.03)	1.17 (1.15–1.18)
Men								
Age (years)								
51–60 (*n* = 752)	1.30 (1.24–1.34)	1.09 (1.06–1.10)	1.13 (1.10–1.16)	1.10 (1.07–1.13)	1.23 (1.19–1.26)	1.15 (1.11–1.16)	1.03 (1.02–1.04)	1.17 (1.14–1.19)
41–50 (*n* = 845)	1.22 (1.17–1.25)	1.08 (1.05–1.09)	1.12 (1.09–1.14)	1.11 (1.07–1.13)	1.23 (1.19–1.25)	1.12 (1.10–1.14)	1.02 (1.01–1.04)	1.14 (1.12–1.16)
31–40 (*n* = 468)	1.14 (1.08–1.17)	1.07 (1.03–1.09)	1.08 (1.05–1.10)	1.08 (1.04–1.10)	1.23 (1.18–1.26)	1.11 (1.08–1.12)	1.01 (1.00–1.02)	1.12 (1.09–1.13)
Professional qualification								
Unskilled/semi-skilled (*n* = 105)	1.36 (1.18–1.45)	1.11 (1.04–1.15)	1.17 (1.09–1.24)	1.13 (1.05–1.20)	1.29 (1.18–1.36)	1.22 (1.12–1.27)	1.00 (1.00–1.00)	1.21 (1.13–1.27)
Occupational training (*n* = 1492)	1.26 (1.23–1.29)	1.09 (1.07–1.10)	1.14 (1.11–1.16)	1.12 (1.09–1.13)	1.24 (1.21–1.25)	1.13 (1.11–1.16)	1.03 (1.02–1.04)	1.16 (1.14–1.17)
University (*n* = 467)	1.11 (1.07–1.14)	1.04 (1.03–1.05)	1.04 (1.02–1.05)	1.03 (1.01–1.04)	1.19 (1.15–1.21)	1.10 (1.07–1.12)	1.01 (1.00–1.02)	1.09 (1.07–1.10)
Disabilities								
Cases (*n* = 188)	1.62 (1.45–1.72)	1.19 (1.12–1.24)	1.31 (1.21–1.38)	1.22 (1.12–1.29)	1.40 (1.31–1.46)	1.18 (1.13–1.23)	1.04 (1.01–1.07)	1.32 (1.24–1.37)
Non-cases (*n* = 1848)	1.19 (1.16–1.20)	1.06 (1.05–1.07)	1.09 (1.07–1.10)	1.08 (1.06–1.09)	1.21 (1.19–1.23)	1.12 (1.11–1.13)	1.02 (1.01–1.03)	1.13 (1.11–1.14)
Congenital Diseases								
Cases (*n* = 96)	1.42 (1.24–1.54)	1.12 (1.05–1.16)	1.18 (1.08–1.24)	1.12 (1.03–1.19)	1.31 (1.20–1.38)	1.16 (1.08–1.20)	1.05 (1.00–1.12)	1.22 (1.14–1.27)
Non-cases (*n* = 1965)	1.23 (1.19–1.24)	1.08 (1.06–1.08)	1.11 (1.09–1.12)	1.10 (1.08–1.11)	1.23 (1.20–1.24)	1.13 (1.11–1.14)	1.02 (1.01–1.03)	1.14 (1.12–1.15)
Accidents								
Cases (*n* = 772)	1.29 (1.23–1.32)	1.10 (1.08–1.12)	1.15 (1.11–1.17)	1.13 (1.09–1.15)	1.27 (1.23–1.29)	1.15 (1.12–1.16)	1.02 (1.00–1.03)	1.18 (1.15–1.19)
Non-cases (*n* = 1289)	1.20 (1.16–1.22)	1.06 (1.05–1.07)	1.10 (1.08–1.11)	1.08 (1.06–1.10)	1.21 (1.18–1.22)	1.12 (1.10–1.13)	1.02 (1.01–1.03)	1.13 (1.11–1.14)

Age was associated with the NFAS total and domain scores of both sexes. As shown in Table 3, a significantly lower functional level became apparent for employees aged 51–60 (women ${\overline{\mathrm{x}}}_W$ =1.23, CI = 1.19–1.25 and men ${\overline{\mathrm{x}}}_M$ =1.17, CI = 1.14–1.19) as compared to those aged 31–40 years (women ${\overline{\mathrm{x}}}_W$ =1.15, CI = 1.11–1.17 and men ${\overline{\mathrm{x}}}_M$ =1.12, CI = 1.09–1.13).

Unskilled/semi-skilled employees showed a significantly higher NFAS total score (women ${\overline{\mathrm{x}}}_W$ =1.29, CI = 1.19–1.36 and men ${\overline{\mathrm{x}}}_M$ =1.21, CI = 1.13–1.27) than employees holding a university degree (women ${\overline{\mathrm{x}}}_W$ =1.15, CI = 1.11–1.17 and men ${\overline{\mathrm{x}}}_M$ =1.09, CI = 1.07–1.10) (Table 3).

Among the 336 disabled individuals, 39% were determined to have a degree of disability of 50% or higher. Individuals with confirmed disabilities were found to have significantly higher NFAS total scores than their counterparts without such condition, which was true for both sexes (women: cases ${\overline{\mathrm{x}}}_W$ =1.47, CI = 1.37–1.53 vs. non-cases ${\overline{\mathrm{x}}}_W$ =1.16, CI = 1.14–1.17; men: cases ${\overline{\mathrm{x}}}_M$ =1.32, CI = 1.24–1.37 vs. non-cases ${\overline{\mathrm{x}}}_M$ =1.13, CI = 1.11–1.14). For both women and men the variable disability revealed the highest NFAS total scores among the five considered variables. Likewise, being disabled resulted in the highest scores of any of the NFAS domains with a maximum of ${\overline{\mathrm{x}}}_W$ =1.80, CI = 1.62–1.91 for women in the domain Walking/standing (Table 3).

Also, there were significant differences in the NFAS total scores between individuals who had an accident (women ${\overline{\mathrm{x}}}_W$ =1.25, CI = 1.21–1.28 and men ${\overline{\mathrm{x}}}_M$ =1.18, CI = 1.15–1.19) and those who hadn’t (women ${\overline{\mathrm{x}}}_W$ =1.17, CI = 1.15–1.18 and men ${\overline{\mathrm{x}}}_M$ =1.13, CI = 1.11–1.14). Concerning congenital diseases there were significant differences in the NFAS total score for women (cases ${\overline{\mathrm{x}}}_W$ =1.33, CI = 1.20–1.39 vs. non-cases ${\overline{\mathrm{x}}}_W$ =1.18, CI = 1.16–1.19) and numerical differences for men (cases ${\overline{\mathrm{x}}}_M$ =1.22, CI = 1.14–1.27 vs. non-cases ${\overline{\mathrm{x}}}_W$ =1.14, CI = 1.12–1.15).

### Combined effects of age, professional education and health limitations on the NFAS

Separate analyses result in raw differences of the mean, which can overestimate the effect of each variable. Therefore, for all single variables described above the effects were estimated simultaneously in a regression model (Table 4).

**Table 4:** Generalised linear regression model of the NFAS total score by sex

	Women (*n* = 1866)		Men (*n* = 2060)	
Variable	Beta coefficients	95% CI	Beta coefficients	95% CI
Intercept	0.558***	0.450–0.665	0.357***	0.286–0.428
Age (years)				
51–60	0.054***	0.027–0.081	0.023*	0.002–0.044
41–50	0.006	−0.018 – 0.031	0.008	−0.011 – 0.028
31–40 (ref.)	0		0	
Professional qualification				
Unskilled/semi-skilled	0.110**	0.043–0.178	0.032	−0.017 – 0.081
Occupational training (ref.)	0		0	
University	−0.027*	−0.049 – −0.005	−0.053***	−0.070 – −0.036
Disabilities vs. no disabilities	0.274***	0.193–0.355	0.165***	0.111–0.220
Congenital diseases vs. no congenital diseases	0.084*	0.008–0.159	0.044	−0.008 – 0.095
Accidents vs. no accidents	0.080***	0.050–0.110	0.031**	0.013–0.050

*p **<** 0.05, **p **<** 0.01, *** *p* < 0.001

For both sexes the regression reconfirmed a significantly lower functional level of the oldest age group, for disabled employees and employees who had an accident. These factors resulted in an increase of the NFAS total score by up to 0.274 points for women and up to 0.165 points for men (disabilities vs. no disability, Table 4). The regression model reconfirmed that female employees with a congenital disease had a significantly higher NFAS total score, expressing a lower functional ability, which was not found for men. For women and men a university degree was significantly associated with a higher functional level referring to the NFAS total scores.

With one exception (unskilled/semi-skilled women vs. women with an occupational training) all simultaneously estimated effects on the NFAS total score (Table 4) were smaller than the single effects given by the mean differences in Table 3. This means that the considered variables have a correlated influence on the NFAS total score. For example, the difference in NFAS total scores between women with and without disabilities was 0.274 points as estimated via regression analysis, whereas the single consideration of ‘disabilities’ vs. ‘no disabilities’ resulted in a mean difference of 0.31 points (Table 3). The reason is that of the 557 women who had at least one health limitation 34 (6.1%) had a combination of disability plus accident, around 3% had a congenital disease plus accident or a congenital disease plus disability, respectively, and less than 1.5% had all three health limitations.

## Discussion

The NFAS was developed as a measure of individual functioning and was applied here to the target population of employees for the first time. Relevant co-factors such as sex, age, professional qualifications, employees that suffer from disabilities, congenital diseases and accidents were considered for the analyses.

Overall, the level of functioning as indicated by a NFAS total score of 1.17 was very high. This positive tendency was further underscored by the fact that 35% of the employees reported the best possible functional ability regarding the 39 queried activities.

Considering single factors, we found that men generally rated a significantly better functioning than women, except for the items of the domains Cooperation/communication and Senses. A better self-reported functional ability of men compared to women was expected on the basis of previous NFAS studies [11–14], as was the well-known age-dependency on functional ability that was found in our study, too. We found positive effects of higher professional education on the NFAS total score, and negative effects in case of being an unskilled or semi-skilled employee. Effects of education as shown previously in [22] and in line with the NFAS literature [11–14] were reconfirmed by the NFAS-results at hand. Employees with disabilities and serious accidents showed significantly more functional problems than their unaffected counterparts – a result that also fits well with previous findings [10, 12]. Additionally, suffering from a confirmed disability revealed the most problems on each of the NFAS domains, and is plausible, well-described and proof of the NFAS scale in itself [23].

Two methods were used to estimate the effects of the factors on the NFAS scores. The first method – calculation of the NFAS scores with confidence intervals for each factor, stratified by sex – revealed slightly higher differences of the NFAS total score for not taking into account co-factors that can be correlated. The second method – running a generalised linear regression with all five co-factors for women and for men – in general resulted in slightly smaller regression coefficients (these coefficients accord to the differences of the NFAS total score). The use of both methods resulted in two effect estimates which provide the range of evidence for normative values of functional ability among employees. Referring to our data, the largest difference of these two effect estimates was 0.04 NFAS points. The important essential is that both methods revealed the same general result: there were more functional difficulties for older employees, females, employees with low professional qualification, and for employees suffering from accidents and confirmed disabilities.

The NFAS score values of the present study can be compared to the Norwegian general population aged 24 to 86 (*n* = 1705) and their NFAS total score of 1.31 [14]. In detail, the Norwegian subgroup of individuals with no sickness absence in the previous year resulted in a NFAS total score of 1.13, as compared to 1.17 for all employees of the German sample and to 1.10 for the German subgroup of always healthy employees (36% of the S-MGA I sample). Among the German sample, 38% had one to 9 days of sick-leave in the year preceding the survey, resulting in a NFAS score of 1.13, just as the very healthy Norwegian subsample. For the remaining third German subsample with 10 or more days of sick-leave a NFAS total score of 1.30 was obtained, exactly replicating the score of the Norwegian individuals that were sick-listed for at least 1 week in the previous year. Very close to these subpopulations ranges the Norwegian general population with their NFAS total score of 1.31. Thus, the combination of age, sickness and the demands of work [2] seem to be key factors for functional ability as measured by the NFAS. Interestingly, the observed pattern of the NFAS domain scores among employees in Germany was very similar to the Norwegian NFAS pattern for almost all NFAS domains: the most severe difficulties appeared for the domains Managing and Walking/standing, the least for the domain Senses with NFAS with values of 1.43, 1.37 and 1.09 for the Norwegian general population and values of 1.26, 1.26 and 1.03 for the employee sample in Germany [14].

Concerning missing data, the study addressed a limitation noted in a previous study [11] by providing the options ‘inapplicable to me’, ‘refused’ and ‘don’t know’. The last two categories were literally irrelevant as they accounted for only 0.01% of the unanswered items. This signifies a high acceptance of the NFAS, potentially further strengthened by having conducted personal interviews instead of questionnaires as in all other applications of the NFAS. The mean level of missing values was similarly low for the Norwegian and the German sample (2.6% and 2.0%, respectively). For further applications of the NFAS, one missing category ‘inapplicable to me’ seems to be sufficient.

To our knowledge, there are no cut-off values which are relevant for a process at the interface of workforce participation and rehabilitation. Having seen that more than one-third of the employees showed the ideal point of 39 times a value of 1, it still remains an open question to determine a maximum level of non-functioning for participating at work. From our perspective, this issue has to be addressed through longitudinal studies, having return-to-work as an outcome.

As a main strength of the study we consider its large sample size based on random sampling within a nationwide sampling frame. Furthermore, this data base provides normative values of the NFAS for a reference population of employees, taking into account sex, age, professional qualification, and health limitations. It takes only 10 min to respond to the items, and the frequency of item non-response appears to have a negligible effect. Hence, it is assumed that the NFAS is highly accepted by employees. With the longitudinal data of the S-MGA survey that will repeatedly include the NFAS, it will be possible to recommend NFAS domains and to find out levels for a premature exit of older employees from the labour market. The present study is limited by the participant group. On the one hand it solely includes persons with a dependent employment, and on the other hand their age is restricted to 31 to 60 years.

## Conclusions

This study presents stratified NFAS scores of a representative sample of employees by age, profession qualification, and for individuals with confirmed disabilities, congenital disorders and accidents. The main findings were that women, elder employees and individuals who are unskilled or semi-skilled reported more functional problems than men, younger employees and persons with a university degree. Employees with disabilities, congenital diseases and accidents had significantly more functional problems than their counterparts without such conditions. Disabled employees showed most functional problems, scoring highest on each of the NFAS domains. While considering all five variables simultaneously, the single effects were reconfirmed. One third of employees reported no difficulty for all the items. Most problems with functional abilities were reported within the domains Walking/standing and managing. The data constitute a basis of normative values of functional ability for the workforce.

## Additional file

Additional file 1: (DOCX 19 kb)

## Abbreviations

CAPI: Computer-assisted personal interview
CI: Confidence interval
ICF: International Classification of Functioning, Disability and Health
NFAS: Norwegian Function Assessment Scale
SD: Standard deviation
S-MGA: Studie zur mentalen Gesundheit bei der Arbeit; Study on Mental Health at Work
SPSS: Statistical Package for the Social Sciences

## References

[1] Kirch W (2008). Encyclopedia of public health: volume 1: A-H volume 2: I-Z.

[2] Nordenfelt L (2008). The concept of work ability.

[3] Badura B, Ducki A, Schröder H, Klose J, Meyer M (2015). Fehlzeiten-Report 2015.

[4] De Boer WEL, Brenninkmeijer W, Zuidam W (2004). Long-term disability arrangement. A comparative study of assessments and quality control.

[5] Statistics Norway (2014). Statistical yearbook of Norway 2013.

[6] Robert Koch-Institut (Hrsg) (2015). Gesundheit in Deutschland. Gesundheitsberichterstattung des Bundes.

[7] Brage S, Donceel P, Falez F (2008). Development of ICF core set for disability evaluation in social security. Disabil Rehabil.

[8] Cerniauskaite M, Quintas R, Boldt C, Raggi A, Cieza A, Bickenbach JE (2011). Systematic literature review on ICF from 2001 to 2009: its use, implementation and operationalisation. Disabil Rehabil.

[9] Organization WH (2001). International classification of functioning, disability and health: ICF.

[10] Brage S, Fleten N, Knudsrød O, Reiso H, Ryen A (2004). Norwegian functional scale - a new instrument in sickness certification and disability assessments. Tidsskrift for den Norske laegeforening: tidsskrift for praktisk medicin, ny raekke.

[11] Osteras N, Brage S, Garratt A, Benth JS, Natvig B, Gulbrandsen P (2007). Functional ability in a population: normative survey data and reliability for the ICF based Norwegian function assessment scale. BMC Public Health.

[12] Magnussen LH, Strand LI, Eriksen HR (2009). Physical and mental functioning in disability pensioners with back pain. J Musculoskelet Pain.

[13] Oyeflaten I, Midtgarden IJ, Maeland S, Eriksen HR, Magnussen LH (2014). Functioning, coping and work status three years after participating in an interdisciplinary, occupational rehabilitation program. Scand J Public Health.

[14] Osteras N, Gulbrandsen P, Garratt A, Benth JS, Dahl FA, Natvig B (2008). A randomised comparison of a four- and a five-point scale version of the Norwegian function assessment scale. Health Qual Life Outcomes.

[15] Schröder H, Schiel S, Schulz S, Kleudgen M (2015). Mentale Gesundheit bei der Arbeit (S-MGA): Methodenbericht zur Repräsentativerhebung an Erwerbstätigen in Deutschland. 2. überarbeitete Auflage ed.

[16] Bundesamt S (2011). Bevölkerung und Erwerbstätigkeit. Haushalte und Familien. Ergebnisse des Mikrozensus.

[17] Rose U, Schiel S, Schröder H, Kleudgen M, Tophoven S, Rauch A (2017). The study on mental health at work: design and sampling. Scand J Public Health.

[18] Bundesamt S (2016). Statistisches Jahrbuch Deutschland und Internationales 2016.

[19] SYSTAT Inc (2009). SYSTAT 13. Statistics I.

[20] Rüschendorf L. Mathematische Statistik. Berlin: Springer; 2014.

[21] Tweedie MCK. An index which distinguishes between some important exponential families. Statistics: Applications and New Directions - Proceedings of the Indian Statistical Institute Golden Jubilee International Conference. Calcutta: Indian Statistical Institute. 1984;579–604.

[22] Grammenos S (2003). Illness, disability and social inclusion.

[23] Falvo D (2014). Medical and psychosocial aspects of chronic illness and disability.

