# Supplementary material for: Application of the ICF based Norwegian function assessment scale to employees in Germany
Source: J Occup Med Toxicol. 2018 Jan 11;13:3. doi: 10.1186/s12995-017-0183-4 (PMC5765624; doi:10.1186/s12995-017-0183-4)
Supplement: Additional file 1: — (DOCX 19 kb) [file 12995_2017_183_MOESM1_ESM.docx]

**Norwegian Function Assessment Scale (NFAS) – German Adaptation**

**Kodierung der Itemkategorien:**

1: Keine Schwierigkeiten

2: Wenige Schwierigkeiten

3: Mäßige Schwierigkeiten

4: Große Schwierigkeiten

5: Konnte ich nicht

**NUR für Interviewer, nicht vorlesen:**

6: trifft nicht zu, Tätigkeit nicht ausgeübt

7: verweigert

8: weiß nicht

**Hatten Sie aus gesundheitlichen Gründen im Laufe der letzten Woche Schwierigkeiten bei der Ausführung dieser Tätigkeiten?**

**Bitte geben Sie mir zu jeder Tätigkeit an, ob Sie keine Schwierigkeiten, wenige Schwierigkeiten, mäßige Schwierigkeiten oder große Schwierigkeiten hatten oder ob Sie die Tätigkeit nicht ausüben konnten.**

**Hatten Sie in der letzten Woche Schwierigkeiten …:**

1. zu stehen
2. weniger als 1 km auf ebener Strecke zu gehen
3. mehr als 1 km auf ebener Strecke zu gehen
4. auf wechselndem Untergrund zu gehen
5. Treppen zu steigen
6. einzukaufen
7. Schuhe und Strümpfe anzuziehen

**Im Folgenden geht es um andere Dinge des Alltags.**

**Bitte geben Sie mir wieder zu jeder Tätigkeit an, ob Sie aus gesundheitlichen Gründen im Laufe der letzten Woche keine Schwierigkeiten, wenige Schwierigkeiten, mäßige Schwierigkeiten oder große Schwierigkeiten hatten oder ob Sie die Tätigkeit nicht ausüben konnten.**

**Hatten Sie in der letzten Woche Schwierigkeiten …:**

1. mit den Fingern eine Münze vom Tisch zu nehmen
2. ein Lenkrad mit den Händen zu halten und zu steuern
3. Auto zu fahren
4. Essen zuzubereiten
5. zu schreiben
6. allgemeine Alltagsaufgaben selbständig auszuführen
7. Ihre Freizeitaktivitäten auszuüben
8. sich an- und auszuziehen

**Die nächsten Fragen beginnen mit Heben oder Tragen von Dingen. Es geht weiterhin darum, ob Sie mit diesen Tätigkeiten aus gesundheitlichen Gründen im Laufe der letzten Woche damit Schwierigkeiten hatten.**

**Hatten Sie in der letzten Woche Schwierigkeiten …:**

1. einen Gegenstand mit dem Gewicht eines Kastens mit leeren Wasserflaschen aus Glas vom Boden hochzuheben
2. Einkaufstaschen mit den Händen zu tragen
3. eine Tasche bzw. Rucksack auf der Schulter oder auf dem Rücken zu tragen
4. mit den Armen zu schieben oder zu ziehen
5. Ihre Wohnung zu putzen
6. Ihre Wäsche zu waschen

**Es folgen nun drei Fragen, bei denen es ums Sitzen geht. Bedenken Sie auch hier wieder, ob Sie aus gesundheitlichen Gründen im Laufe der letzten Woche Schwierigkeiten hatten oder nicht.**

**Hatten Sie in der letzten Woche Schwierigkeiten …:**

1. auf einem Küchenstuhl zu sitzen
2. in einem Auto als Beifahrer mitzufahren
3. öffentliche Verkehrsmittel zu benutzen

**Abschließen möchte ich Ihnen weitere Tätigkeiten vorlesen, über die wir noch nicht gesprochen haben. Denken Sie bitte wieder an die letzte Woche und ob Sie diese Tätigkeiten aus gesundheitlichen Gründen ausführen konnten.**

**Hatten Sie in der letzten Woche Schwierigkeiten …:**

1. aufmerksam und konzentriert zu sein
2. in einer Gruppe zu arbeiten
3. andere bei deren Aktivitäten anzuleiten
4. alltäglichen Pflichten nach zu kommen
5. die alltäglichen Herausforderungen und Belastungen zu bewältigen
6. Kritik auszuhalten
7. Zorn und Aggressionen zu kontrollieren
8. sich zu erinnern
9. mündliche Mitteilungen zu verstehen
10. schriftliche Mitteilungen zu verstehen
11. zu sprechen
12. an Gesprächen mit mehreren Personen teilzunehmen
13. das Telefon zu benutzen
14. Fernsehen zu schauen
15. Radio zu hören
